# Correction: Identification of one critical amino acid that determines a conformational neutralizing epitope in the capsid protein of porcine circovirus type 2

**DOI:** 10.1186/s12866-023-03077-4

**Published:** 2023-10-28

**Authors:** Li P Huang, Yue H Lu, Yan W Wei, Long J Guo, Chang M Liu

**Affiliations:** Division of Swine Infectious Diseases, State Key Laboratory of Veterinary Biotechnology, Harbin Veterinary Research Institute, The Chinese Academy of Agricultural Sciences, 427 Maduan Street, Nangang District, Harbin, 150001 China

Correction: *BMC Microbiol* **11**, 188 (2011)

10.1186/1471-2180-11-188

Following publication of the original article [1], the authors identified errors in Figs. 2 and 3a, and 5. The correct figures are given below. The authors apologize for any inconvenience. This correction does not affect the results or the conclusion of this work.

**Fig. 2:**
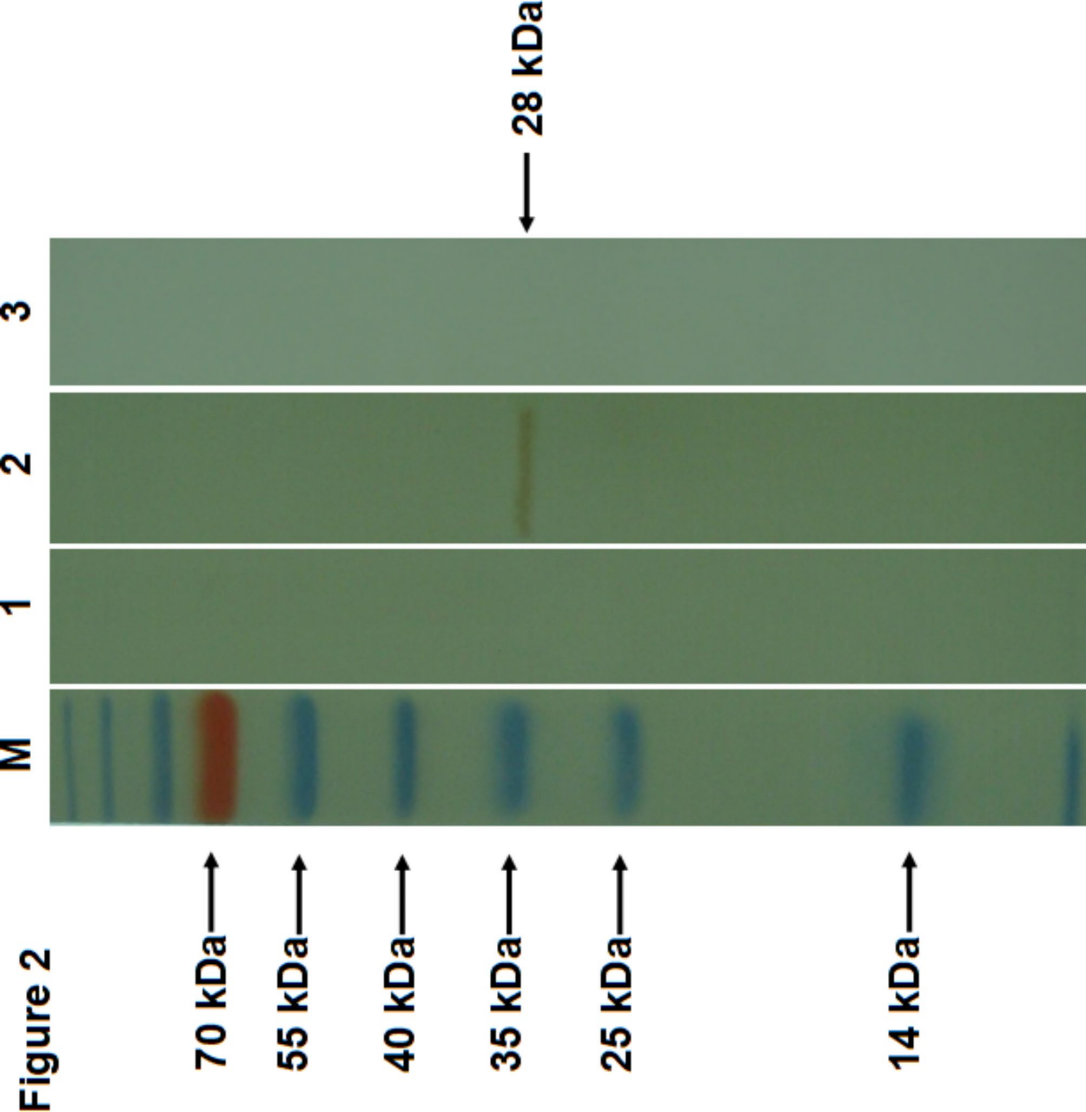
Analysis of immunoreactivity of mAb by western blot analysis. Purified virions of the PCV2a/LG strain were separated by SDS-PAGE, transferred to nitrocellulose membranes, and incubated with mAb. Lane M: protein molecular weight markers; lane 1: mAb 8E4; lane 2: mAb 6F10 as a positive control; lane 3: SP2/0 supernatant as a negative control

**Fig. 3:**
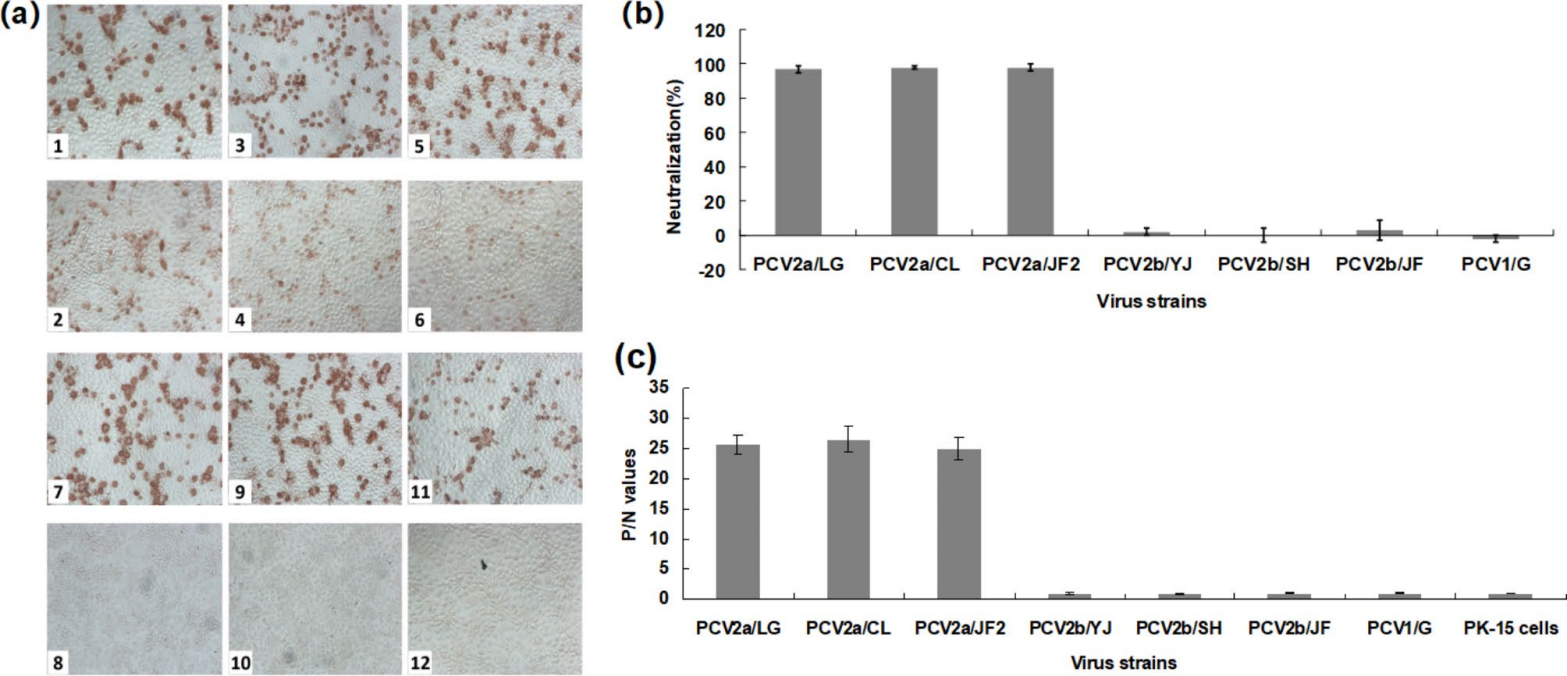
Reactivity of six PCV2 isolates with mAb 8E4 by the IPMA, serum neutralization assay and capture ELISA. (**a**) IPMA reactivity of PK-15 cells inoculated with PCV2a/LG (1 and 2), PCV2a/CL (3 and 4), PCV2a/JF2 (5 and 6), PCV2b/YJ (7 and 8), PCV2b/SH (9 and 10) and PCV2b/JF (11 and 12), against PCV2-positive serum and mAb 8E4. Odd numbers represent PCV2-positive serum, whereas even numbers show mAb 8E4. (**b**) The neutralizing activity of mAb 8E4 was expressed as the percentage reduction in the number of infected cells in comparison with negative control. A mean neutralizing activity of > 50% was considered to represent neutralization. Error bars represent the standard deviations. (**c**) For the capture ELISA, cultures of six PCV2 isolates, recPCV1/G and PK-15 cells were tested with HRP-conjugated 8E4. P/N > 2.1 was regarded as a positive result. Error bars represent the standard deviations

**Fig. 5:**
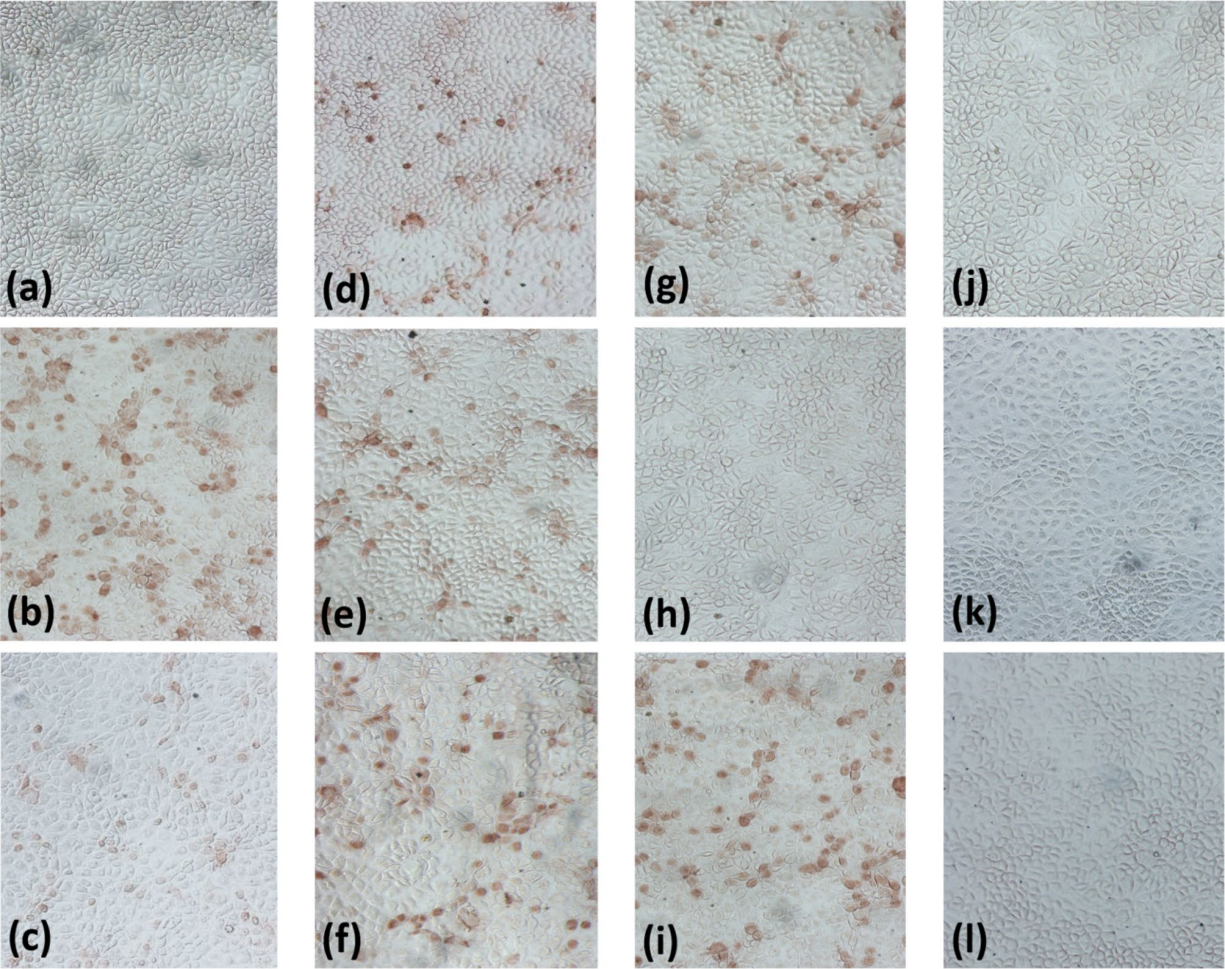
IPMA reactivity between mAb 8E4 and each chimera or mutant. (**a**) rCL-YJ-1; (**b**) rCL-YJ-2; (**c**) rCL-YJ-3; (**d**) rCL-YJ-4; (**e**) rCL-YJ-5; (**f**) rCL-YJ-1-51; (**g**) rCL-YJ-1-57; (**h**) rCL-YJ-1-59; (**i**) rCL-YJ-1-63; (**j**) rLG-YJ-1-59; (**k**) rJF2-YJ-1-59; (**l**) rYJ-CL-1-59
